# Does Inclusion of Thickened Liquids in a Modified Barium Swallow Study (MBSS) Protocol Affect DIGEST Grades?

**DOI:** 10.1007/s00455-025-10874-8

**Published:** 2025-10-23

**Authors:** Ryan J. Burdick, Jenni Wu, Ella Aldridge, Claire Terp, Sara Gustafson, Joanne Yee, Carla Warneke, Nicole Rogus-Pulia, Katherine Hutcheson

**Affiliations:** 1 Department of Medicine, Division of Geriatrics and Gerontology, University of Wisconsin-Madison, 1685 Highland Avenue, 5158 Medical Foundation Centennial Building, Madison, WI 53705-2281, USA; 2 Department of Communication Sciences and Disorders, University of Wisconsin-Madison, 1975 Willow Dr, Madison, WI 53706, USA; 3 Geriatrics Research, Education, and Clinical Center (GRECC), Madison Veterans Affairs (VA) Hospital, 2500 Overlook Terrace, Madison, WI 53705, USA; 4 School of Medicine and Public Health, Graduate Program of Clinical Investigation, University of Wisconsin, Madison, USA; 5 Department of Biostatistics, University of Texas MD Anderson Cancer Center, 1155 Pressler St, Houston, TX 77030, USA; 6 Department of Head and Neck Surgery, University of Texas MD Anderson Cancer Center, 1155 Pressler St, Houston, TX 77030, USA; 7 Division of Radiation Oncology, University of Texas MD Anderson Cancer Center, 1155 Pressler St, Houston, TX 77030, USA

**Keywords:** DIGEST, Thickened liquids, Assessment, Videofluoroscopy, Dissemination and implementation

## Abstract

The Dynamic Imaging Grade of Swallowing Toxicity (DIGEST) is a psychometric tool used during modified barium swallow studies (MBSSs) to grade swallowing safety, efficiency, and overall pharyngeal swallow function ranging from 0 (no impairment) to 4 (life-threatening). DIGEST was originally validated on a protocol without routine inclusion of thickened liquids. To ensure stability of DIGEST in different settings, we aimed to assess whether DIGEST grades remained stable when derived from a bolus protocol with or without standard inclusion of thickened liquids. MBSSs from 118 unique participants were retrospectively analyzed by 4 reliable raters using a master protocol of thin, thickened, pudding, and solid boluses. DIGEST grades were derived from four bolus protocol conditions: (1) DIGEST core protocol (without thickened liquids); (2) core plus mildly thick liquid; (3) core plus moderately thick; and (4) core plus mildly and moderately thick. Prevalence and Bias-Adjusted Kappa (PABAK) values were calculated to determine agreement of DIGEST grades between the core protocol and variations. PABAK ranged from 0.83 to 1.00 (near-perfect to perfect agreement). In rare instances where thickened liquids affected grades, they invariably worsened by one grade. There was no clear change-driving bolus type for this effect. Safety was more susceptible to change than efficiency. Inclusion of thickened liquids does not appear necessary in a minimum bolus protocol for DIGEST. Clinicians and researchers who wish to routinely include thickened liquids in their protocol should be aware that DIGEST grades may be worsened by one in a minority of cases and that safety grades appear more likely to be affected than efficiency grades.

## Introduction

The Dynamic Imaging Grade of Swallowing Toxicity (DIGEST) is a comprehensive tool for psychometric assessment of functional swallowing outcomes (i.e., swallowing safety and efficiency). The tool was developed by Hutcheson and colleagues in 2017 [1] and was designed specifically to be commensurate with the National Cancer Institute Common Terminology Criteria for Adverse Events (CTCAE). As such, the tool yields swallowing safety (S) and efficiency (E) grades of 0–4, which correspond to a range of “no impairment” to “life-threatening impairment.” These grades are then synthesized into one overall DIGEST (D) grade of the same 0–4 range.

The DIGEST tool was originally validated in a clinical study from the originating institution (MD Anderson Cancer Center) following a standardized bolus protocol consisting of 10 boluses in lateral plane, including six trials of thin liquid (two 5 mL, two 10 mL, two unregulated cup sips), two trials of pudding, and two trials of a solid cracker bolus. While a standard protocol is needed for initial validation of a tool, it is also valuable to understand the stability of results when a protocol is subject to reasonable changes. This is clear given that the reality of everyday clinical practice and the inherent variability between patients present challenges to consistent protocol adherence.

One such reasonable protocol change is the routine inclusion of thickened liquids. Use of thickened liquids is frequent in clinical practice [2], and is included in some validated bolus protocols [3], but excluded from others [1]. Thickened liquids may reveal impairments or differences in swallow function that would otherwise not be found. For instance, thickened liquids have been shown to acutely reduce airway invasion [4–6], influence physiological factors that may impact swallowing efficiency, such as base of tongue retraction [7], and have rheological properties that change bolus flow through the pharynx [8]. Conversely, a lack of responsiveness to thickened liquids may reveal a greater degree of safety impairment than would have been assumed by testing only thin liquids. However, this possibility currently remains unexplored specific to DIGEST grading.

As a first step to determine whether thickened liquids are crucial to a minimal DIGEST protocol, we aimed to determine the agreement of DIGEST grades (i.e., S, E, and D grades) collected from protocols inclusive of thickened liquids compared to the same protocol without thickened liquids. In the event of any disagreement between protocol variations, we aimed to identify the sources, or drivers, of those changes in relation to bolus consistency, bolus volume, and DIGEST parameter (i.e., S or E). We hypothesized that modified protocols inclusive of thickened liquids would show near-perfect agreement with the protocol that does not include thickened liquids. Furthermore, we hypothesized that, when overall DIGEST grades were not in agreement between protocol variations, 20 mL of mildly thick liquid would be the change driving bolus, and that there would not be a clear change-driving parameter between S and E grades.

## Methods

### Study Design

This retrospective study was conducted under the regulation of an institutional review board (IRB, MD Anderson PA19–0261) with a waiver of informed consent for image review. We utilized baseline data collected from 118 modified barium swallow studies (MBSS) conducted on patients with dysphagia of varying severities as well as healthy controls as part of two previously IRB-approved prospective research studies at the University of Wisconsin-Madison, and the William S. Middleton Memorial Veterans Hospital.

Study 1 was a prospective clinical trial conducted on individuals with very mild to moderate Alzheimer’s Disease and Related Dementias (ADRD) and associated dysphagia; individuals were recruited in dyads with their care partner. Demographic data for participants recruited to study one can be found in Table 1. Inclusionary criteria for participants in Study 1 included the following: (1) Age 50–99, (2) Englishspeaking, (3) Diagnosis of dementia or cognitive impairment or memory loss, (4) Actively involved caregiver, (5) Resides at home, in an assisted living facility, or other longterm care facility, (6) a Clinical Dementia Rating (CDR) score between 0.5 (very mild) and 2 (moderate) [9], (7) Able to undergo a MBSS, (8) Presence of at least one swallow yielding a Penetration-Aspiration Scale (PAS) [10] score 3 or higher, or a Modified Barium Swallow Impairment Profile (MBSImP) [3] score of 2 or higher on the “Pharyngeal Residue” component (i.e., component 16). Inclusion Criteria for Caregivers included: (1) Age 18 or older, (2) English speaking, (3) Contact with the patient at least once per week, (4) Has access to a working telephone. Exclusionary criteria for participants in Study 1 included: (1) Dementia due to cerebrovascular disease as primary cause, (2) History of head and neck cancer or other structural deformity that can affect swallowing, (3) Allergy to barium sulfate, (4) Currently breastfeeding, pregnant or planning to become pregnant. Exclusionary criteria for caregivers in Study 1 only included lack of ability to consent. Individuals were only enrolled in Study 1 if both the participant and their caregiver met the above criteria.

Study 2 was a three-site, prospective, observational study conducted on community-dwelling adult individuals with a wide range of dysphagia etiologies as well as healthy controls. More demographic information regarding these participants can be found in Table 1. Inclusionary criteria for dysphagic participants in Study 2 included: (1) Age 18–99, (2) Dysphagia as determined by a licensed speech pathologist via MBSS, (3) Candidacy for, and agreement to participate in, a strength-based dysphagia treatment program. Inclusionary criteria for control participants in Study 2 included: (1) Age 18–99, (2) No swallowing impairment seen on MBSS as determined by a licensed speech pathologist. Exclusionary criteria for both dysphagic and control participants in Study 2 included: (1) Inability to provide consent, (2) History of allergic response to barium sulfate, and (3) History of allergic response to topical anesthetic (Study 2 included the completion of high-resolution pharyngeal manometry).

All participants successfully recruited to both studies at the time of analysis were included in this retrospective study as long as their modified barium swallow study (MBSS) included the presentation of at least one thickened liquid. MBSSs had been previously de-identified, and consequently were not traceable back to the original study participants. MBSSs were recorded and acquired at 30 frames/pulses per second.

### DIGEST Ratings

In total, four trained and reliable (i.e., a minimum 80% agreement during a training period) raters were used to derive DIGEST grades of Safety (S), Efficiency (E), and overall impairment (D) on the above 118 MBSSs. Ratings were conducted on Image J [11] using frame-by-frame analysis. All S, E, and D grades were calculated in duplicate by two of the four raters. Subsequent to the initial ratings, all discrepancies in S, E, and D grades were resolved via a consensus process in which at least two raters reviewed MBSSs and agreed upon a final rating. All MBSSs were completed with a master protocol, a portion of which resembled the original DIGEST validation bolus protocol (i.e., six thin (International Dysphagia Diet Standardisation Initiative (IDDSI) 0) boluses, two Varibar pudding (IDDSI 4) boluses, and two solid (graham cracker; IDDSI 7) boluses, subsequently referred to as the core protocol. While the core protocol was similar to the DIGEST validation protocol, key differences included the following: (1) One less thin liquid bolus presentation (one 10 mL bolus as opposed to two), (2) replacement of two unregulated cup sips of thin liquid with two 20 mL thin liquid presentations. In addition to the core protocol, the master protocol also included a single 5 mL mildly thick (IDDSI 2) bolus, a single 20 mL mildly thick bolus, and (only in the 24 patients from study #1) a single 5 mL moderately thick (IDDSI 3) bolus. DIGEST grades were calculated four times based on four different protocol variations including: (1) DIGEST core protocol (no thick liquids), (2) DIGEST core plus mildly thick liquids (i.e., Core + Mild), (3) DIGEST core plus moderately thick liquids (i.e., Core + Mod), and (4) DIGEST core plus mildly and moderately thick liquids (i.e., Core + Mild + Mod). These four protocol variations can also be viewed in Table 2.

### Data Analysis

Prevalence and bias-adjusted (weighted) kappa (PABAK) [12, 13] values were calculated to determine levels of agreement between the DIGEST core bolus protocol and the three variations listed above in regard to S, E, and D grades. Further, descriptive statistics were taken from all videos showing discrepancies in S, E, D grades between any of the protocol variations to determine the drivers of change in regard to consistency, volume, and parameter.

## Results

### DIGEST Distributions

**Fig. 1:**
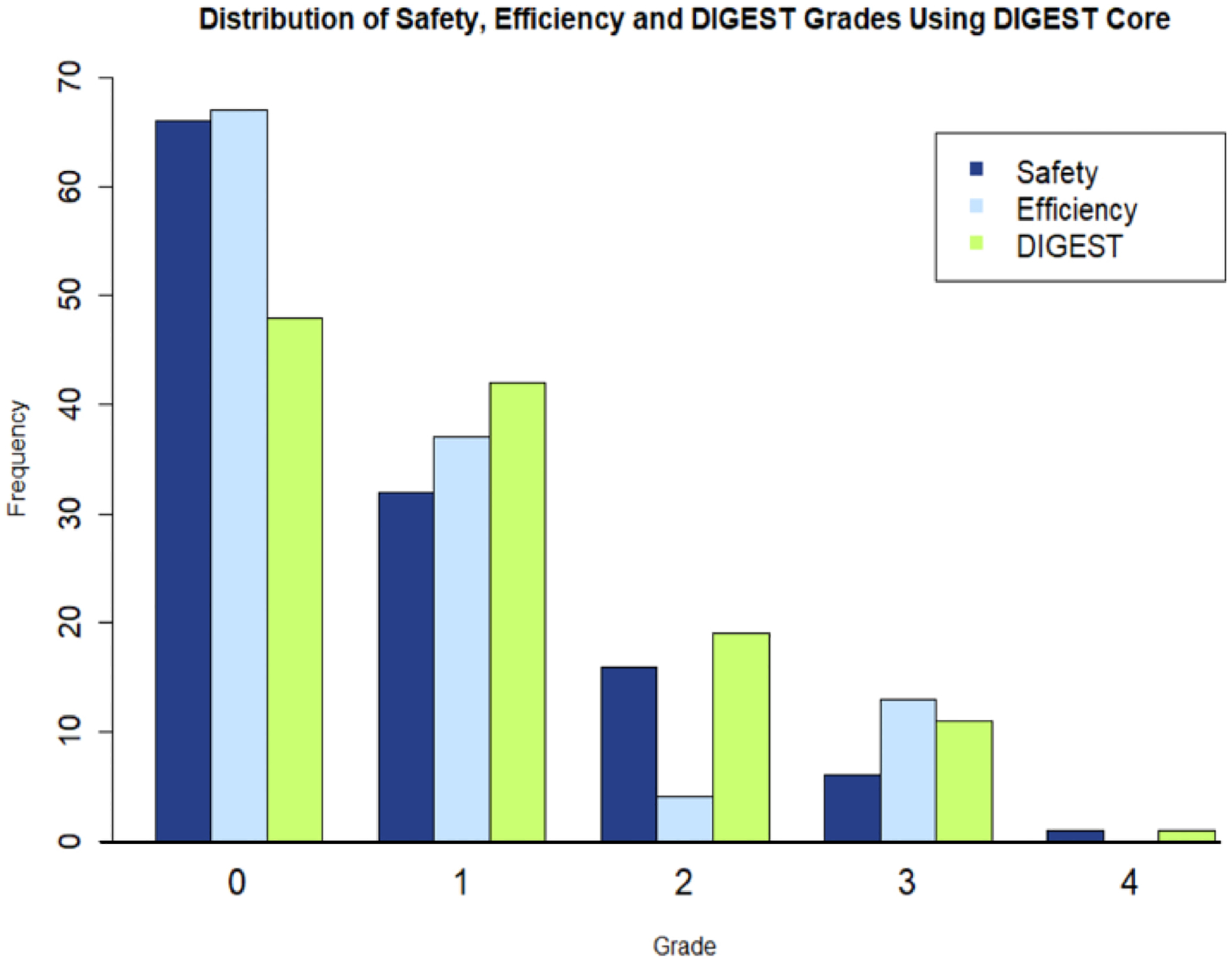
A bar plot showing the distributions of Safety (S), Efficiency (E), and Overall DIGEST (D) grades derived from the DIGEST core protocol alone

DIGEST ratings for the core MBS protocol can be seen in Fig. 1. The distribution of S, E, and D grades seen in this work is commensurate with previous studies employing this tool [14, 15]. MBSSs from a total of 118 unique individuals were rated for analysis. Of these studies, all 118 included mildly thick liquids, allowing for the calculation of DIGEST + Mildly thick liquids grades, and 24 studies included moderately thick liquids, allowing for the calculation of 24 DIGEST + Moderately thick liquids and 24 DIGEST + Mild + Moderately thick liquid grades. As such, a total of 284 Safety, Efficiency, and Overall DIGEST grades were calculated.

### Percent Agreement

Contingency tables showing overall agreement between the DIGEST core protocol and the three protocol variations can be seen in Table 3. Percent agreement between protocol variations ranged from 92 to 100%. When discrepancies occurred between the core protocol and the modified protocols inclusive of thickened liquids, DIGEST grades (i.e., S, E, and D grades) invariably worsened by one grade.

### Prevalence and Bias-Adjusted Kappa

Prevalence and bias-adjusted kappa values comparison S, E, and D grades between the three protocols inclusive of thickened liquids and the DIGEST core protocol can be viewed in Table 4. Kappa values ranged from 0.83 to 1, corresponding to near-perfect to perfect agreement [16].

### Drivers of Change

#### Consistency and Volume

Of the 498 total comparisons made between S, E, and D grades across protocol variations (354 for DIGEST Core vs. Core + Mild; 72 for DIGEST Core vs. Core + Mod; and 72 for DIGEST Core vs. Core + Mild + Mod), 484 (97%) of these comparisons were in perfect agreement for S, E, D grades. Of the 14 (3%) discrepancies noted, there was not a consistent change-driving consistency or volume. When the core protocol was compared to the core + mildly thick protocol, the change-driving boluses included 5 mL mildly thick (2/10; 20%), 20 mL mildly thick (5/10; 50%), and both 5 mL and 20 mL mildly thick (3/10; 30%) across S, E, and D grades. When the core was compared to core + moderately thick, the 5 mL moderately thick bolus elicited one instance of change (S grade). Finally, when the core was compared to core + mildly thick + moderately thick, the drivers of change were 5 mL mildly thick (2/3; 66.6%), and 5 mL moderately thick (1/3; 33%) across S, E, and D grades.

#### DIGEST Parameter

Of the 166 comparisons in overall DIGEST grade (i.e., D grade), 160 (96%) of these comparisons were in perfect agreement. Of the remaining six (4%) comparisons revealing a discrepancy, Safety (S) grades were the driver of that change in five instances (83.3%).

## Discussion

This study is part of a larger clinical implementation project (R01271223) focused on facilitating real-world clinical use of DIGEST in diverse practice settings. One of the fundamental factors necessary to ensure that DIGEST can be used reliably in different practices is understanding how it performs under different MBSS acquisition standards. As such, our goal is to ensure DIGEST results are stable with different standard bolus protocols—focusing this work on thickened liquids. Herein, we aimed to determine the level of agreement between DIGEST grades acquired from an MBSS bolus protocol that is exclusive of thickened liquids, compared to the same protocol with thickened liquids routinely included. The results of this work suggest that consistent inclusion of thickened liquids in an MBSS protocol rarely affects DIGEST grades (eliciting change in < 5% of cases), as near-perfect to perfect agreement was achieved between the core protocol and all of the protocol variations tested. As such, in the pursuit of defining a minimum protocol to clinically implement DIGEST grading, thickened liquids are likely not needed in every examination. In other words, impairments specifically to *safety* and *efficiency* observed on thickened liquids are generally also captured with administration of the thin liquid and solid trials in the standardized bolus protocol used in the validation of DIGEST. It is also important to note that these results were obtained in a general dysphagic population that is not specific to head and neck cancer, thus increasing the generalizability of these results.

Routinely testing thickened liquids only changed 4% of DIGEST grades, and this was almost always an increase in the Safety grade. These results appear counter-intuitive initially, given that thickened liquids have been shown to generally reduce the likelihood of airway invasion [5, 17–19]. The most recent and rigorous efforts examining the effects of bolus type on swallow safety come from Borders and Steele [17], who performed a Bayesian analysis on a dataset of 855 individuals, revealing that thin liquids elicit the highest likelihood of penetration-aspiration. Results of this work and similar studies may lead individuals to incorrectly suspect that DIGEST safety grades could not be worsened with inclusion of thickened liquids. However, reduced responsiveness to the therapeutic effects of thickened liquids as a means to reduce aspiration of liquids may reflect an increased degree of impairment. This logic was incorporated into the DIGEST decision tree wherein DIGEST safety grading considers the pattern of higher-grade PAS grades (PAS ≥ 3) across IDDSI-defined bolus levels; depending on the PAS pattern with thin liquids, a PAS score(s) of 5 or higher on thick liquids may increase the DIGEST Safety grade. The rarity of this observation in our dataset, however, supports our clinical experience that the DIGEST safety grade is almost always driven by the pattern of PAS on thin liquids (in > 95% of examinations in this dataset). It is also important to note that while standardly testing thickened liquids occasionally isolated a small subset of individuals who demonstrate airway invasion of thin liquids *and* mildly thick liquids, the majority of individuals in this study exhibited the expected presentation of improved swallow safety with thickened liquids compared to thin liquids.

Considering the structure of the DIGEST, it is important to highlight that inclusion of thickened liquids does not allow for the possibility of improved DIGEST grades. Rather, grades can only be conserved or worsened. Psychometrically, the DIGEST is designed to take the worst score obtained across all boluses presented, and then add context to that worst score based on the surrounding pattern over which that ‘worst’ impairment occurred [1, 20]. For Efficiency grades, these patterns account for the type of bolus on which the worst score was achieved (i.e., liquid (IDDSI 1–3), pureed/pudding (IDDSI 4), or solid (IDDSI 7)). For Safety grades, the pattern is established by considering both the bolus type (airway invasion on single or multiple consistencies), the frequency of airway invasion on any single consistency—usually thin liquids (single event, less than 50% of trials *of one consistency*, or greater than or equal to 50% of trials *of one consistency*), and finally the volume of invasion (trace, > 25% of the bolus, or neither). If the worst score is obtained on thin liquid boluses, then adding thickened liquids will not change the DIGEST grade. Likewise, if a thickened liquid bolus elicits one of the worst ratings already seen on another bolus type, this can only worsen or maintain DIGEST grades. In these cases, safety grades have the potential to be worsened either by increasing the number of consistencies on which airway invasion is observed (i.e., higher frequency of worst PAS), or by eliciting a greater volume of airway invasion on thickened liquids (i.e., higher volume of worst PAS). Meanwhile, efficiency grades would only worsen if thickened liquids elicited a greater residue percentage compared to thin liquids.

The results of this study should not be misinterpreted to imply that the results of thick liquid bolus trials should be ignored in DIGEST. In fact, since conception, DIGEST operating rules have instructed the rater to consider the results of thick liquid trials when they are administered in scoring the DIGEST result. This decision may seem counter to the goal of standardization but was thoughtfully considered. The rationale was multifactorial. First, DIGEST was designed to be a pragmatic tool used in a clinical setting wherein ad hoc administration of thick liquid almost universally implies that there is impairment in swallow safety that merits further testing across bolus levels. Ignoring the thick liquid trials in the pattern of swallow safety threatens to diminish the ecological validity of the DIGEST result. Secondly, in our development of the tool for cancer populations, there were sub-populations for whom thick liquids were more frequently score driving (e.g., patients treated with partial laryngectomy or patients with severe/profound grade 3–4 dysphagia and early bolus protocol deviation). For these reasons, users choosing to implement thickened liquids ad hoc in their bolus protocol should include these results in DIGEST grading.

Expanding upon the results of this work, this study has implications for the minimal protocol needed when deriving DIGEST grades. While the use of a standardized protocol is necessary for initial validation of a tool, rigidity in a bolus protocol may present limitations to successful dissemination and implementation of psychometric tools. As practice tools such as DIGEST are implemented in real-world clinics, it is important to understand how stable the instrument is when used with different imaging protocols. There is a growing body of research acknowledging that the consistent use of standard protocols while conducting modified barium swallow studies is limited in clinical practice [21, 22]. This is expected, given the unpredictable nature of clinical practice, and the consistent need to tailor the instrumental assessment of swallowing physiology and function to the needs of the patient. The requirement for flexibility in a MBSS protocol was upheld in a recent expert panel, which stated that a standardized protocol does not imply “rigidity” or “inflexibility”, but rather the acknowledgement that a minimum protocol is necessary to fully understand the integrity of the swallowing mechanism [23]. While a minimum DIGEST protocol is still in development, these results reveal that thickened liquids in most cases are inconsequential to DIGEST grades, and thus, do not appear necessary in a minimum protocol for DIGEST.

## Limitations

This work is not without its limitations. While the results of this study are more clear for protocols inclusive of mildly thick liquids, moderately thick liquid observations were only available on a small subset of individuals, all with Alzheimer’s disease and dysphagia. As such, the question of whether standardized inclusion of moderately thick liquids can affect DIGEST grades requires further inquiry performed on a larger sample. Additionally, it is noted that the protocol variations did not involve the presentation of repeated bolus trials; as such, it is possible that additional trials may affect DIGEST score beyond what was observed in this study.

Further, while effort was made to match the bolus protocol used in the original DIGEST validation study as closely as possible, there were logistical limitations that did not allow the core protocol to match the one originally used during validation. In comparison to the original validation protocol, the core protocol in our lab excluded one 10 mL thin bolus and included 20 ml sips of thin liquid in lieu of unregulated cup sips. While not an exact match to the original DIGEST validation protocol, our core protocol was still within the DIGEST authors’ prior recommendations, with the authors stating in their most recent validation that “a minimum standard bolus protocol necessary to give high confidence in valid DIGEST results should include 5 to 6 uncompensated thin liquid bolus trials” [20].

## Future Directions

These results provide important future directions for this line of inquiry. The results observed in this study are only applicable to the DIGEST used during a MBSS. Consequently, it will be important to determine whether these results also generalize to the DIGEST used during flexible endoscopic evaluation of swallowing (DIGEST-FEES) [24]. Additionally, thickened liquids are not the only protocol modification that could reasonably occur during the modified barium swallow study; the use of sequential swallowing tasks are noted in other protocols [25], and present unique challenges to the swallowing mechanism that would be of consequence in a minimal DIGEST protocol [26]. Further, given that the psychometric parameters of the DIGEST are only designed to evaluate swallowing *safety* and *efficiency*, these results do not provide insights into the utility of thickened liquids in revealing impairments in swallowing *physiology* on MBSS. Prior studies have been conducted examining this with thickened liquids eliciting differences in various aspects of the Modified Barium Swallow Impairment Profile (MBSImP) [7] as well as temporal aspects of swallowing, including swallow reaction time and timing of maximum pharyngeal constriction relative to opening of the upper esophageal sphincter [6]. The results of this study do not contradict these findings highlighting differences in swallowing physiology based on bolus consistency, and further inquiry on this topic will continue to benefit the field.

## Conclusions

The results of this study suggest that standardized inclusion of thickened liquids in an MBSS protocol generally does not affect DIGEST grades. However, on the rare occasion that thickened liquids do alter DIGEST grades, one can expect a worsening of grade by one category, with the parameter of Safety being the most likely source of change.

## Figures and Tables

**Table 1:** A table representing participant demographics for study 1, study 2, and the composite of these studies for the retrospective analysis completed

VARIABLE	*n*			
	STUDY 1	STUDY2		TOTAL
Dysphagia Status	Dysphagic	Dysphagic	Non Dysphagic	
*n*	26	56	36	**118**
*Average Age*	76.7	69.9	61.4	**69.3**
(σ, Range)	(9.1; 55–91)	(9.32; 44–90)	(12.0; 34–84)	**(12.2; 34–91)**
*Biological Sex*				
Male	17 (65.4%)	55 (98.2%)	33 (91.7%)	**105 (89.0%)**
Female	9 (34.6%)	1 (1.8%)	3 (8.3%)	**13 (11.0%)**
*Gender*				
Male	17 (65.4%)	55 (98.2%)	32 (88.9%)	**104 (88.1%)**
Female	9 (34.6%)	1 (1.8%)	4 (11.1%)	**14 (11.9%)**
*Primary Dysphagia Etiology*				
Dementia	26 (100%)	0 (0%)	NA	**26 (31.7%)**
Gastroesophageal	0 (0%)	11 (19.6%)	NA	**11 (13.4%)**
Progressive Neurological Disorder	0 (0%)	9 (16.1%)	NA	**9 (11.0%)**
Head and Neck Cancer	0 (0%)	8 (14.3%)	NA	**8 (9.8%)**
Cervical Spine	0 (0%)	8 (14.3%)	NA	**8 (9.8%)**
Respiratory Disorder	0 (0%)	6 (10.7%)	NA	**6 (7.3%)**
Stroke	0 (0%)	4 (7.1%)	NA	**4 (4.9%)**
Connective Tissue	0 (0%)	2 (3.6%)	NA	**2 (2.4%)**
Cardiac	0 (0%)	1 (1.8%)	NA	**1 (1.2%)**
General	0 (0%)	1 (1.8%)	NA	**1 (1.2%)**
Deconditioning/Weakness	0 (0%)	4 (7.1%)	NA	**4 (4.9%)**
Etiology Not Specified	0 (0%)	2 (3.6%)	NA	**2 (2.4%)**

**Table 2:** A table representing the four various protocol iterations (Core, Core + Mildly Thick (Mild), Core + Moderately Thick (Mod), Core + Mild + Mod) pulled from the master protocol used to conduct this study

Amount (mL)	Consistency	IDDSI Level	Number of Trials	Protocol Variation			
				Core	Core + Mild	Core + Mod	Core + Mild + Mod
5	Thin	0	2				
10	Thin	0	1				
20	Thin	0	2				
5	Puree	4	2				
¼ Cracker + 3mL Puree	Solid	7	2				
5	Mildly thick	2	1				
20	Mildly thick	2	1				
5	Moderately thick	3	1				

Filled cells represent inclusion of the mentioned boluses in the protocol iteration, while blank cells represent exclusion

**Table 3:** Three contingency tables comparing safety (S), efficiency (E), and overall DIGEST (D) grades derived from the core protocol compared to core + Mildly Thick (top), core + Moderately Thick (middle) and core + Mildly + Moderately Thick (bottom)

DIGEST CORE VS CORE + MILDLY THICK (IDDSI 2)																
Safety (S) Percent Agreement: 96% (113/118)																
Efficiency (E) Percent Agreement: 99% (117/118)																
Overall DIGEST (D) Percent Agreement: 97% (114/118)																
		DIGEST CORE														
		0			1			2			3			4		
		S	E	D	S	E	D	S	E	D	S	E	D	S	E	D
DIGEST CORE + MILDLY THICK	0	**62**	**66**	**46**	*0*	*0*	*0*	*0*	*0*	*0*	*0*	*0*	*0*	*0*	*0*	*0*
	1	3	1	2	**28**	**35**	**39**	*0*	*0*	*0*	*0*	*0*	*0*	*0*	*0*	*0*
	2	*0*	*0*	*0*	2	*0*	1	**16**	**4**	**17**	*0*	*0*	*0*	*0*	*0*	*0*
	3	*0*	*0*	*0*	*0*	*0*	*0*	*0*	*0*	1	**6**	**12**	**11**	*0*	*0*	*0*
	4	*0*	*0*	*0*	*0*	*0*	*0*	*0*	*0*	*0*	*0*	*0*	*0*	**1**	**0**	**1**
DIGEST CORE VS CORE + MODERATELY THICK (IDDSI 3)																
Safety (S) Percent Agreement: 96% (23/24)																
Efficiency (E) Percent Agreement 100% (24/24)																
Overall DIGEST (D) Percent Agreement: 100% (24/24)																
		DIGEST CORE														
		0			1			2			3			4		
		S	E	D	S	E	D	S	E	D	S	E	D	S	E	D
DIGEST CORE + MODERATELY THICK	0	**13**	**15**	**9**	*0*	*0*	*0*	*0*	*0*	*0*	*0*	*0*	*0*	*0*	*0*	*0*
	1	*0*	*0*	*0*	**5**	**4**	**7**	*0*	*0*	*0*	*0*	*0*	*0*	*0*	*0*	*0*
	2	*0*	*0*	*0*	*0*	*0*	*0*	**2**	**0**	**4**	*0*	*0*	*0*	*0*	*0*	*0*
	3	*0*	*0*	*0*	*0*	*0*	*0*	1	*0*	*0*	**3**	**5**	**4**	*0*	*0*	*0*
	4	*0*	*0*	*0*	*0*	*0*	*0*	*0*	*0*	*0*	*0*	*0*	*0*	**0**	**0**	**0**
DIGEST CORE VS CORE + MILDLY (IDDSI 2) +MODERATELY THICK (IDDSI 3)																
Safety (S) Percent Agreement: 92% (22/24)																
Efficiency (E) Percent Agreement 100% (24/24)																
Overall DIGEST (D) Percent Agreement: 100% (24/24)																
		DIGEST CORE														
		0			1			2			3			4		
		S	E	D	S	E	D	S	E	D	S	E	D	S	E	D
DIGEST CORE + MILDLY + MODERATELY THICK	0	**13**	**15**	**9**	*0*	*0*	*0*	*0*	*0*	*0*	*0*	*0*	*0*	*0*	*0*	*0*
	1	*0*	*0*	*0*	**4**	**4**	**7**	*0*	*0*	*0*	*0*	*0*	*0*	*0*	*0*	*0*
	2	*0*	*0*	*0*	1	*0*	*0*	**2**	**0**	**3**	*0*	*0*	*0*	*0*	*0*	*0*
	3	*0*	*0*	*0*	*0*	*0*	*0*	1	*0*	1	**3**	**5**	**4**	*0*	*0*	*0*
	4	*0*	*0*	*0*	*0*	*0*	*0*	*0*	*0*	*0*	*0*	*0*	*0*	**0**	**0**	**0**

Bolded cells represent those in which the protocols were in agreement

**Table 4:** Table showing prevalence and bias-adjusted kappa values with 95% confidence intervals comparing safety (S), efficiency (E), and overall DIGEST (D) grades derived from the core protocol compared to core + Mildly Thick (Mild), core + Moderately Thick (Mod), and core + Mild + Mod

DIGEST Core +	Safety	Efficiency	Overall DIGEST
Mildly thick (IDDSI 2; *n* = 118)	**0.92** (0.81–0.97)	**0.98** (0.91–0.99)	**0.93** (0.83–0.98)
Moderately thick (IDDSI 3; *n* = 24)	**0.92** (0.58–0.99)	**1** (0.72–1)	**1** (0.72–1)
Mildly + Moderately thick (IDDSI 2 & 3; *n* = 24)	**0.83** (0.46–0.98)	**1** (0.72–1)	**0.92** (0.58–0.99)

Bold indicate the actual PABAK values as opposed to the 95% confidence intervals

## Data Availability

Given that the data associated with this work is medical in nature, the analytical dataset will be made available upon reasonable request to the Senior Author and after full execution of a data use agreement with our institution.

## References

[1] Hutcheson KA, Barrow MP, Barringer DA, Knott JK, Lin HY, Weber RS, Fuller CD, Lai SY, Alvarez CP, Raut J, Lazarus CL, May A, Patterson J, Roe JW, Starmer HM, Lewin JS. Dynamic imaging grade of swallowing toxicity (DIGEST): scale development and validation. Cancer. 2017;123(1):62–70. 10.1002/cncr.30283. 27564246 PMC5161634

[2] Cichero JA, Lam P, Steele CM, Hanson B, Chen J, O Dantas R, Development of international terminology and definitions for texture-modified foods and thickened fluids used in dysphagia management: the IDDSI framework. Dysphagia. 2017. 10.1007/s004550169758y. PMC5380696 27913916

[3] Martin-Harris B, Brodsky M, Michel Y, O Castell D, Schleicher M, Sandidge J, Maxwell R, Blair J. MBS measurement tool for swallow impairment–MBSImp: establishing a standard. Dysphagia. 2008. 10.1007/s0045500891859. PMC4217120 18855050

[4] Newman R, Vilardell N, Clave P, Speyer R. Effect of bolus viscosity on the safety and efficacy of swallowing and the kinematics of the swallow response in patients with oropharyngeal dysphagia: white paper by the European Society for Swallowing Disorders (ESSD). Dysphagia. 2016. 10.1007/s0045501696968. PMC4929168 27016216

[5] Logemann JA, Gensler G, Robbins J, Lindblad AS, Brandt D, Hind JA, A randomized study of three interventions for aspiration of thin liquids in patients with dementia or Parkinson’s disease. J Speech Lang Hear Res. 2008. 10.1044/10924388(2008/013). PMC2894528 18230864

[6] Steele C, Peladeau-Pigeon M, Barbon C, Guida B, Namasivayam-MacDonald A, Nascimento W, Reference values for healthy swallowing across the range from thin to extremely thick liquids. J Speech Lang Hear Res. 2019. 10.1044/2019_JSLHRS180448. PMC6808317 31021676

[7] Garand KL, Armeson K, Hill EG, Blair J, Pearson W, Martin-Harris B. Quantifying oropharyngeal swallowing impairment in response to bolus viscosity. Am J Speech Lang Pathol. 2024;33(1):460–7. 10.1044/2023_AJSLP2300082 37902448 PMC11001168

[8] Hadde EK, Cichero JA, Zhao S, Chen W, Chen J. The importance of extensional rheology in bolus control during swallowing. Scientific Reports. 2019;9(1):16106. 10.1038/s41598019522694 31695062 PMC6834566

[9] Morris JC. Clinical dementia rating: A reliable and valid diagnostic and staging measure for dementia of the alzheimer type. Int Psychogeriatr. 1997;Suppl 1:173–6. 10.1017/s1041610297004870. 9447441

[10] Rosenbek JC, Robbins JA, Roecker EB, Coyle JL, Wood JL. A penetration-aspiration scale. Dysphagia. 1996. 10.1007/BF00417897. 10467048

[11] Schneider CA, Rasband WS, Eliceiri KW. NIH image to imageJ: 25 years of image analysis. Nat Methods. 2012;9(7):671–5. 10.1038/nmeth.2089. 22930834 PMC5554542

[12] Byrt T, Bishop J, Carlin J. Bias, prevalence and kappa. J Clin Epidemiol. 1993;46:423–9. 10.1016/08954356(93)90018V. 8501467

[13] Chen G, Faris P, Hemmelgarn B, Walker RL, Quan H. Measuring agreement of administrative data with chart data using prevalence unadjusted and adjusted kappa. BMC Med Res Methodol. 2009;9:5. 10.1186/1471228895. 19159474 PMC2636838

[14] Tabor LC, Plowman EK, Vasilopoulos T, Gallastegui A. Airway sensorimotor function in individuals with amyotrophic lateral sclerosis. Amyotroph Lateral Scler Frontotemporal Degener. 2018;19:332. 10.1080/21678421.2018.1510582. PMC6513719 30652513

[15] Noorani M, Bolognone RK, Graville DJ, Palmer AD. The association between dysphagia symptoms, DIGEST scores, and severity ratings in individuals with Parkinson’s disease. Dysphagia. 2023;38(5):1295–307. 10.1007/s00455023105554. 36692654

[16] McHugh ML. Interrater reliability: the kappa statistic. Biochem Med (Zagreb). 2012;22(3):276–82. 23092060 PMC3900052

[17] Borders JC, Steele CM. The effect of liquid consistency on penetration-aspiration: a bayesian analysis of two large datasets. Front Rehabil Sci. 2024. 10.3389/fresc.2024.1337971. PMC10920265 38463609

[18] Masuda H, Ueha R, Sato T, Goto T, Koyama M, Yamauchi A, Risk factors for aspiration pneumonia after receiving liquid-thickening recommendations. Otolaryngol Head Neck Surg. 2022;167(1):125–32. 10.1177/01945998211049114. 34582292 PMC9251747

[19] Bülow M, Olsson R, Ekberg O. Videoradiographic analysis of how carbonated thin liquids and thickened liquids affect the physiology of swallowing in subjects with aspiration on thin liquids. Acta Radiol. 2003;44(4):366–72. 10.1080/j.16000455.2003.00100.x. 12846685

[20] Hutcheson KA, Barbon CEA, Alvarez CP, Warneke CL. Refining measurement of swallowing safety in the dynamic imaging grade of swallowing toxicity (DIGEST) criteria: validation of DIGEST version 2. Cancer. 2022;128(7):1458–66. 10.1002/cncr.34079. 34985765 PMC8917062

[21] Benfield JK, Michou E, Everton LF, Mills C, Hamdy S, Bath PM, The landscape of videofluoroscopy in the UK: a web-based survey. Dysphagia. 2021;36(2):250–8. 10.1007/s00455020101301. 32417980 PMC8004508

[22] Power M, Laasch H-U, Kasthuri RS, Nicholson DA, Hamdy S. Videofluoroscopic assessment of dysphagia: a questionnaire survey of protocols, roles and responsibilities of radiology and speech and language therapy personnel. Radiography. 2006;12(1):26–30. 10.1016/j.radi.2005.03.003.

[23] Martin-Harris B, Canon CL, Bonilha HS, Murray J, Davidson K, Lefton-Greif MA. Best practices in modified barium swallow studies. Am J Speech Lang Pathol. 2020;29(2s):1078–93. 10.1044/2020_ajslp1900189. 32650657 PMC7844340

[24] Starmer HM, Arrese L, Langmore S, Ma Y, Murray J, Patterson J, Pisegna J, Roe J, Tabor-Gray L, Hutcheson K. Adaptation and validation of the dynamic imaging grade of swallowing toxicity for flexible endoscopic evaluation of swallowing: DIGESTFEES. J Speech Lang Hear Res. 2021;64(6):1802–10. 10.1044/2021_jslhr2100014. 34033498

[25] Martin-Harris B, Brodsky MB, Michel Y, Castell DO, Schleicher M, Sandidge J, MBS measurement tool for swallow impairment–MBSImp: establishing a standard. Dysphagia. 2008;23(4):392–405. 10.1007/s0045500891859. 18855050 PMC4217120

[26] Ambrocio KR, Miles A, Bhutada AM, Choi D, Garand KL. Defining normal sequential swallowing biomechanics. Dysphagia. 2023;38(6):1497–510. 10.1007/s0045502310576z. 37097448 PMC11554329

